# Case Report: Treatment of delayed tremor episodes in a patient with DPPX antibody encephalitis

**DOI:** 10.3389/fimmu.2025.1594823

**Published:** 2025-07-30

**Authors:** Miao Yu, XinSheng Liu, JunQiang Yan

**Affiliations:** ^1^ Department of Neurology, The First Affiliated Hospital, College of Clinical Medicine of Henan University of Science and Technology, Luoyang, China; ^2^ Key Laboratory of Neuromolecular Biology, The First Affiliated Hospital of Henan University of Science and Technology, Luoyang, China

**Keywords:** anti-dipeptidyl-peptidase-like protein 6 (DPPX), autoimmune encephalitis, tremor, blurred vision, headache

## Abstract

**Introduction:**

Autoimmune encephalitis is a neurological disease caused by abnormal autoimmune mechanisms, characterized by a range of symptoms such as psychiatric and behavioral abnormalities, cognitive impairment, memory decline, and seizures. It is primarily identified by the presence of autoantibodies against neuronal surface antigens in the cerebrospinal fluid. This disease is relatively rare in clinical settings, and its diagnosis remains challenging, with fewer than a hundred cases reported to date. Particularly, cases of Anti-DPPX encephalitis presenting with delayed myoclonus and blurred vision are extremely rare. This case report emphasizes the complexity of the diagnosis and the effective treatment of Anti-DPPX encephalitis with delayed myoclonus.

**Case description:**

The patient experienced intermittent fever accompanied by severe headaches for one month, with headaches worsening in an upright position, followed by two hours of vomiting, with stomach contents being expelled. Upon admission, the preliminary diagnosis included suspected central nervous system infection and suspected autoimmune encephalitis. Despite receiving anti-infective and antiviral treatments, as well as acid suppressant and gastric protection therapies, the patient’s condition continued to deteriorate. Both computed tomography (CT) and magnetic resonance imaging (MRI) showed no apparent abnormalities. Further cerebrospinal fluid and serum tests revealed the presence of anti-DPPX antibodies, confirming the diagnosis of Anti-DPPX encephalitis. The patient underwent a comprehensive treatment regimen, including high-dose steroid pulse therapy, intravenous immunoglobulin, antiviral and anti-infective therapy, as well as acid suppressant and gastric protection treatments. Significant symptom improvement was observed, and by the 8th day of hospitalization, the condition had stabilized. In the 8th month of follow-up, the patient suddenly developed persistent tremor in both hands, without obvious cause. without a recurrence of fever or consciousness disturbances. Steroid therapy was restarted in combination with eculizumab, which was later switched to ofatumumab treatment. The patient’s symptoms improved compared to before, and re-examination showed DPPX antibody titers had turned negative. Two months post-discharge, follow-up continued, and the patient’s family reported that the tremors persisted, affecting daily life and studies.

**Conclusions:**

The current patient is the first reported case of Anti-DPPX encephalitis presenting with delayed tremor accompanied by blurred vision. The patient’s condition was quite fluctuating, which led us to discuss the diversity of symptoms as related to extrapyramidal and occipital lobe damage caused by immune-mediated inflammation. Symptom improvement was achieved through a combination of IVMP, eculizumab, and ofatumumab treatments. The prodromal symptoms of Anti-DPPX encephalitis are easily misdiagnosed as infectious diseases due to the heterogeneity of its clinical manifestations. Early identification of the antibody and initiation of immunotherapy can improve the prognosis.

## 1 Introduction

**Figure 1:**
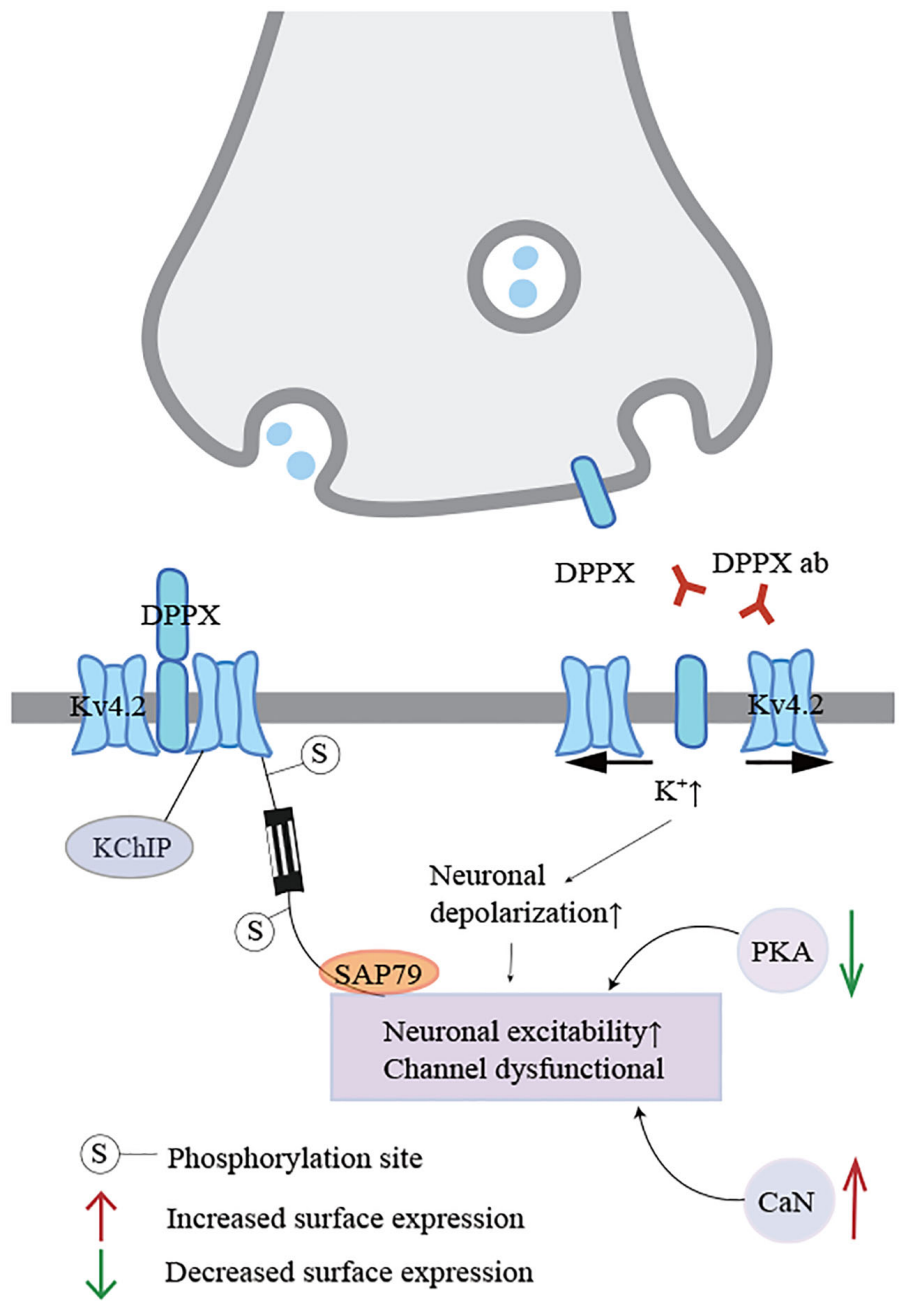
Regulation and dysfunction of Kv4.2 potassium channel complexes. When DPPX is bound by antibodies, the integrity of the Kv4.2-DPPX complex is disrupted, and the potassium channel functions abnormally, linking with the kinase pathway through electrophysiological changes, resulting in uncontrolled neuronal excitability.

Autoimmune encephalitis (AE) is a group of non-infectious diseases mediated by antibodies targeting neuronal surface and synaptic proteins, receptors, and ion channels (1–3), and it is typically responsive to immunotherapy. Dipeptidyl peptidase-like protein (DPPX) is widely distributed in the central nervous system, particularly in the hippocampus, cerebellum, and enteric nervous plexus (4, 5), antibodies against DPPX can lead to abnormal neuronal excitability because DPPX is a regulatory subunit of the voltage-gated Kv4.2 potassium channel complex (6)(**Figure 1**). Anti-DPPX encephalitis (DPPX-AE) is a relatively rare form of AE. It was first reported by Boronat and colleagues in 2013, who identified DPPX-AE (6). Common clinical manifestations often include initial symptoms such as weight loss and diarrhea, which frequently precede central nervous system symptoms such as cognitive dysfunction or memory impairment. Notably, symptoms of central nervous system hyperexcitability, including muscle spasms, tremors, or seizures, should not be overlooked (7, 8). Of course, these symptoms do not necessarily appear simultaneously during the progression of the disease. Providing active treatment in the early stages of the disease can alleviate its progression to some extent, but the appearance of other symptoms is inevitable. Immunotherapy is widely used in most patients with DPPX-AE. Employing proactive immunotherapy during the acute or early phases of the disease has been recognized as effective for most patients (9, 10).Therefore, due to the heterogeneity of clinical symptoms and signs, the diagnosis and treatment of DPPX-AE remain challenging. Here, we report a case study analyzing the risk of delayed tremors in DPPX-AE, as well as the patient’s response and outcomes following several immunotherapy strategies. This provides new suggestions and insights for the diagnosis and treatment strategies of this disease.

## 2 Case description

A 16-year-old Han Chinese girl was admitted to the emergency department due to intermittent fever over the past month, with the highest temperature reaching 38°C, accompanied by a history of headache(mRS:1; CASE:1). Subsequently, she developed repeated vomiting, with vomit consisting of stomach contents, and she visited a tertiary hospital where she vomited approximately 10 times. There is also abdominal pain, excessive sweating, and palpitations, but denies diarrhea or weight loss. The patient reported no loss of consciousness or seizures. She is a young female with no prior history of diabetes, heart disease, hypertension, kidney disease, cerebrovascular disease, or other common medical conditions. Additionally, she has no history of infectious diseases like hepatitis or tuberculosis, nor any history of surgery, blood transfusion, trauma, poisoning, or known allergic reactions to food or medication. She does not smoke or drink alcohol. Furthermore, there is no history of exposure to industrial toxins, dust, or radioactive substances. Currently, known autoimmune diseases, central nervous system infections, epilepsy, and related conditions have been ruled out. The patient’s family, up to three generations, deny any hereditary disease history.

**Figure 2:**
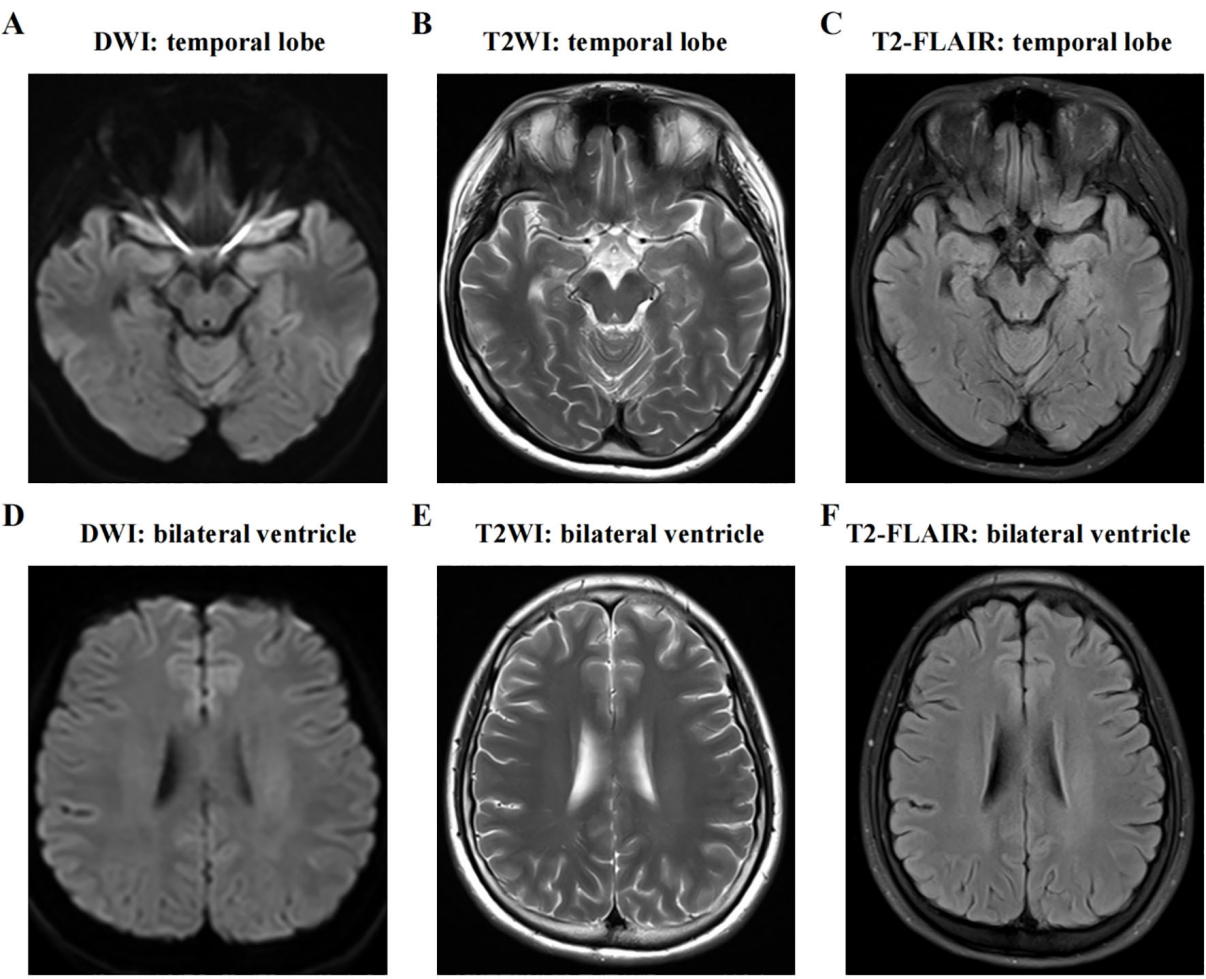
MRI of the brain shows no abnormal signs. The two hemispheres of the brain are symmetrical, with clear distinction between gray matter and white matter. No obvious abnormal signal is detected on axial T1WI, T2WI, FLAIR, or DWI sequences. **(A-C)** T1WI, T2WI, and T2-FLAIR axial view presenting medial temporal lobe; **(D-F)** T1WI, T2WI, and T2-FLAIR axial view presenting lateral ventricle. DWI, diffusion weighted imaging; T2WI, T2-weighted imaging; T2-FLAIR, T2-weighted fluid attenuated inversion recovery.

Upon initial examination, the patient’s temperature was 36.8°C, her speech was fluent, and her pupils were equal in size and round, approximately 3mm in diameter, with sensitive light reflexes. Both eyes were agile in movement without nystagmus, and her limb muscle strength and tone were normal. Her neck was supple without resistance, and she tested positive for the bilateral Babinski sign but negative for Chaddock’s sign and meningeal irritation signs. Abnormal laboratory indicators were recorded as follows: procalcitonin 0.05 ng/ml (<0.046 ng/ml), alkaline phosphatase 37 U/L (117–390 U/L), lymphocyte percentage LYM% 13.9 (20-50%), total T lymphocyte percentage 54.35 (62.6-80.4%), B lymphocyte percentage 28 (11.9-21%), antinuclear antibody (ANA, <1:100), rheumatoid factor (RF, <20), cytomegalovirus (CMV) antibody negative, Epstein-Barr virus (EBV) nucleic acid quantification (5.0×10^2 IU/mL), alpha-fetoprotein (AFP, 0–15 ng/ml), carcinoembryonic antigen (CEA, 0–5 ng/ml), carbohydrate antigen (CA199, 0.1–27 U/L), carbohydrate antigen (CA153, 0.1–25 U/ml), and carbohydrate antigen (CA125, 0.1–35 U/ml), all within the normal reference range. Brain CT and MRI showed no obvious abnormalities (**Figure 2**). Upon admission, her preliminary diagnoses included gastropathy, suspected central nervous system infection, and suspected encephalitis. She was treated with ganciclovir (5mg/kg, once every 12 hours for 2 weeks) for antiviral therapy. Unfortunately, the patient’s condition did not show significant improvement.

**Figure 3:**
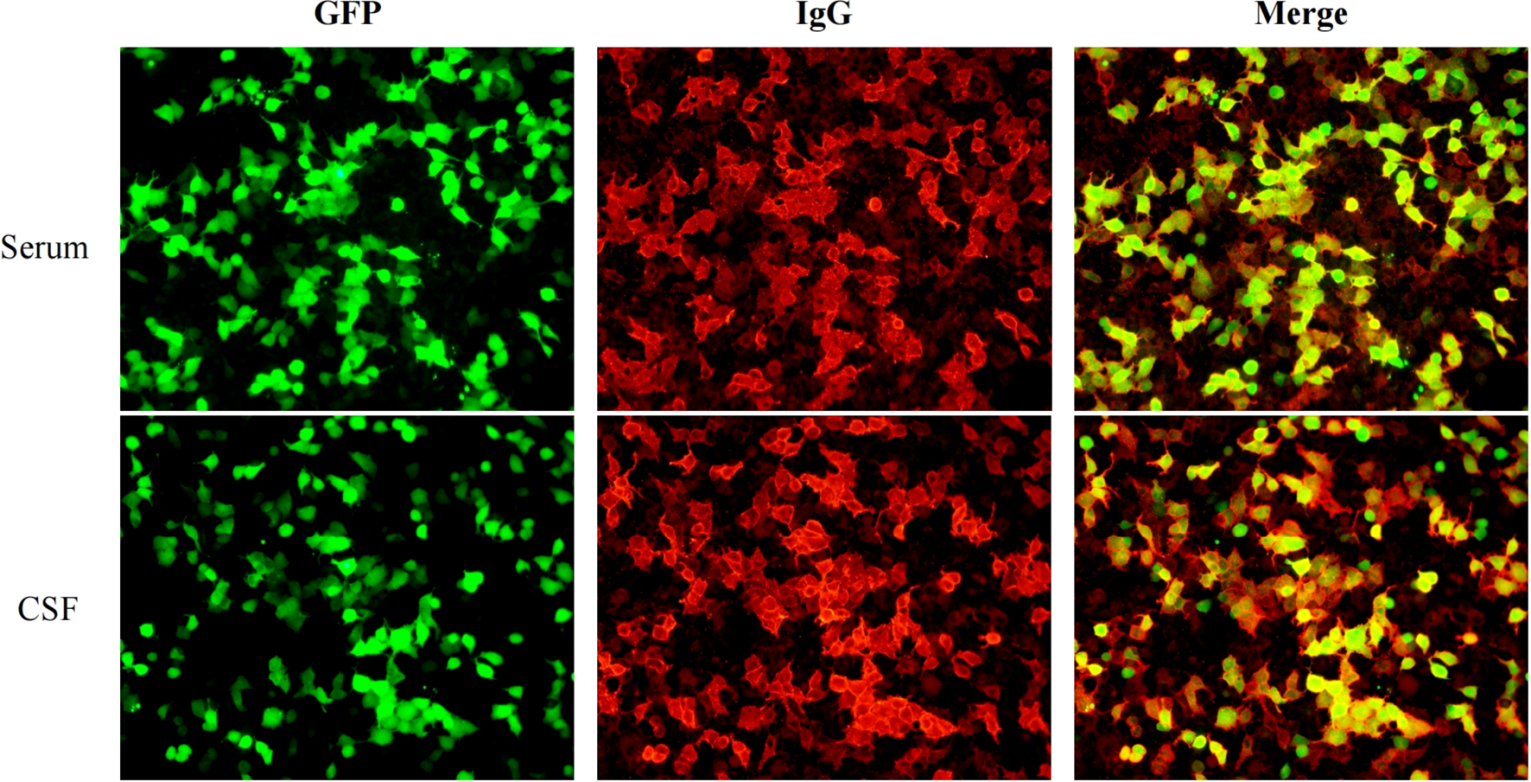
The results of autoimmune encephalitis antibody examination in cerebrospinal fluid and serum by indirect immunofluorescence cell-based assay (CBA-IIF).

On the second day of admission, a lumbar puncture was performed to analyze cerebrospinal fluid (CSF), including routine analysis, biochemical tests, acid-fast staining, and India ink staining. CSF routine analysis revealed a white blood cell count of 0.144 × 10^9^/L (normal range: 0–0.01 × 10^9^/L). CSF biochemical analysis showed a glucose level of 2.76 mmol/L (normal range: 2.8–4.5 mmol/L) and a protein level of 1155 mg/L (normal range: 150–450 mg/L). CSF and serum samples were collected to test for antibodies associated with AE, including anti-NMDA, anti-AMPA1, anti-AMPA2, anti-LGI1, anti-CASPR2, anti-GABAa, anti-GABAb, anti-IGLON5, DPPX, anti-mGluR1, anti-mGluR5, anti-GAD65, anti-MOG, anti-GFAP, and anti-AQP4 antibodies (IgG). The results showed the presence of anti-DPPX antibodies in both CSF and serum, with a serum titer of 1:320 and a CSF titer of 1:100 (**Figure 3**). On the third day of admission, referring to the criteria for autoimmune encephalitis proposed by Graus et al. in 2016 (11), based on clinical presentation, epidemiological characteristics, laboratory results, and brain CT/MRI findings, definite AE, specific disease is Anti-DPPX encephalitis.

The patient’s treatment plan involved high-dose methylprednisolone sodium succinate pulse therapy (3, 11, 12). Specifically, consider the patient as a young female, treated with methylprednisolone sodium succinate using a daily dosage of 500mg for pulse therapy over 3 days, followed by a reduced dosage of 250mg daily for another 3 days. Subsequently, maintenance therapy includes oral administration of prednisone acetate (60mg with tapering). The patient’s condition should be closely monitored for any changes, with continued observation of body temperature variations, and symptomatic interventions should be promptly implemented as needed, to maintain bodily balance and stability.

During the treatment course from day 4 to day 10, the dosage of corticosteroids was gradually reduced. The patient demonstrated significant improvement, with body temperature progressively controlled and stabilized. By the 8th day of hospitalization, the patient’s condition was stable, there was no recurrence of fever, and her mental state was good. Oral corticosteroid therapy continued. After discharge, the patient continued tapering the oral corticosteroids until discontinuation(mRS:0; CASE:0). A 3-month follow-up showed improvement in the patient’s condition. However, in the 8th month of follow-up, the patient suddenly developed persistent tremor in both hands, without obvious cause. Clinical observation recorded a tremor frequency of 6–8 Hz. The tremor is persistent and exacerbates with tension. Importantly, it lacks target directivity, indicating that it does not significantly worsen during visually guided movements, such as the finger-to-nose test. Accompanied by blurred vision, but there was no fever or recurrence of cognitive impairment(mRS:2; CASE:4; TRS:31).

The patient sought medical attention at a tertiary hospital. Abnormal test results were as follows: Interleukin-6 (IL-6): 7.37pg/ml (≦5.4pg/ml), Interleukin-12P70: 6.61pg/ml (≦3.2pg/ml), absolute NKT cell count: 35 cells/μl (45–296 cells/μl), and kappa light chain: 1.69g/L (1.7–3.7g/L). A cranial MRI showed no abnormalities. EEG Results: Findings did not show epileptiform discharges, photoparoxysmal responses, or other abnormalities suggestive of cortical myoclonus generators. Abnormal: Right H-reflex waveform absent. Lumbar puncture for CSF testing, including routine, biochemical, and cytological examination, yielded the following results: CSF white blood cells: 8 × 10^6^/L (≦8 × 10^6^/L), CSF protein: 1.126g/L (0.15–0.45g/L), and Pandy test: positive. CSF and serum samples were tested for anti-DPPX antibodies, which were negative. During this period, a chest CT scan revealed a tiny ground-glass nodule in the right upper lung, likely inflammatory, with a recommendation for dynamic observation. Density in both thyroid lobes was mildly heterogeneous, and ultrasound correlation was recommended. Whole-body lymph node ultrasound showed no abnormalities in the bilateral cervical, axillary, or inguinal regions.

For treatment, the patient received anti-inflammatory corticosteroids along with gastric protection, potassium supplementation, and calcium supplementation. At the same time, efgartigimod (10mg/kg once a week for two weeks) was administered, but the patient’s symptoms did not improve significantly. Later, the treatment regimen included ofatumumab (20mg, the first dose, the second dose every other week, and the third dose every four weeks, the patient only had two shots). After discharge, a 3-month follow-up was conducted(mRS:1; CASE:4; TRS:31). The patient’s family reported that the tremors continued to impact daily life and studies. A timeline of the patient’s diagnostic and treatment process is provided (**Table 1**).

**Table 1:** The patient’s treatment and diagnostic timeline.

Time	2024-05-14	2024-05-15	2024-05-16	2024-05-24	Post-discharge review	2025-01-20	2025-01-23	Post-discharge review
Clinical features	Fever 36.8°C up to 38°C, headache, vomiting, sweating, palpitation	Fever 37.0°C, headache, sweating, palpitation	Temperature 36.6°C, headache, sweating, palpitation	Symptoms improved and no new positive signs were found.	3 months 6 months Status: No recurrence	Persistent tremor of both hands; Blurred vision	Persistent tremor of both hands; Blurred vision	The patient’s family reported that the tremors continued to impact daily life and studies
Accessory examination	Brain CT and MRI: No abnormal signs.	CSF pathogen detection: culture, ink staining, TB. AE antibody: serum & CSF.	CSF pathogen detection: negative. AE antibody: serum & CSF: DPPX antibody positive.	leave hospital	Brain MRI: No abnormal signs.	Brain MRI: No abnormal signs.	AE antibody: serum & CSF: DPPX antibody negative.	
	mRS: 1;CASE:1				mRS: 0; CASE:1	mRS: 2;CASE:4;TRS:31		mRS:1;CASE:4; TRS:31
Treatment	Ganciclovir (5mg/kg, once every 12 hours for 2 weeks); Large dose methylprednisolone sodium succinate pulse therapy 500mg for 3 consecutive days; 250mg for 3 consecutive days, and then oral prednisone acetate 60mg gradually reduced until discontinued.			Continue oral prednisolone acetate outside the hospital and gradually reduce the dose until discontinuation.		Sequential Steroid Treatment (IVMP for 3 days); Efgartigimod (10mg/kg once a week for two weeks); Ofatumumab (20mg, the first dose, the second dose every other week, and the third dose every four weeks, the patient only had two shots).		1.Consider starting maintenance immunotherapy. 2.Referral to a rehabilitation center for movement disorders is recommended.
Diagnose	Gastropathy; Suspected CNS infection; Suspected encephalitis.		DPPX-AE			DPPX-AE		
Patient Perspective	The patient and their family have gone from a lack of understanding of the disease to providing positive feedback during monthly follow-ups.					After the second recurrence, the patient and his family were depressed and uncooperative, and the symptoms did not match the results of auxiliary examination. These factors led to poor effect of the second hospitalization. Despite difficulties, we still tried our best to achieve the best result.		

mRS, ModifiedRankinScale; Fahn, TRS, Tolosa, Marin Tremor Rating Scale; CASE, Clinical Assessment Scale for Autoimmune Encephalitis.

mRS: 1, Although there are symptoms, there is no significant impairment and all daily duties and activities can be performed.

mRS: 2, Mild disability, unable to perform all pre-disease activities but does not require assistance and can manage his own affairs.

TRS: 31, moderate tremor, suggesting a significant impact on daily life.

CASE: 3, mild impairment, suggestive of basic independence.

## 3 Discussion

DPPX-AE commonly involves infratentorial regions (brainstem/cerebellum), where irreversible damage can occur rapidly (13). In DPPX-AE, CSF is typically characterized by inflammation. The disease is typified by a triad of symptoms, including weight loss, central nervous system hyperexcitability, and cognitive deficits. However, recent reports suggest that clinical manifestations may be more heterogeneous. While blood and CSF analyses usually show normal cell counts, high titers of DPPX antibodies in the blood and CSF can confirm DPPX-AE. Xiao et al. conducted a retrospective analysis of nine patients with DPPX-AE. They described three patients with anti-DPPX encephalitis who experienced sleep disorders and abnormal sleep behaviors, accompanied by rapid eye movements, limb paralysis, and severe CSF pleocytosis. One patient, who underwent treatment with methylprednisolone and a trial of tacrolimus, demonstrated significant improvement with no relapses during a 6-month follow-up period (14). The patient’s headache is significantly associated with body position, which may reflect autonomic dysregulation (15). Bjerknes et al. reported a case of a 63-year-old female patient with DPPX- AE, whose initial symptoms included severe limb pain, diarrhea, and weight loss, later progressing to cognitive impairment. Blood and CSF tests revealed strongly positive DPPX antibodies (with gastrointestinal diseases and tumors ruled out). The patient achieved complete recovery after one year of treatment with methylprednisolone combined with rituximab (16). The Kv4.2 potassium channel mediates the A-type current in neurons of the spinal dorsal horn, and its gene deletion leads to increased neuronal excitability, tactile/thermal hypersensitivity, and elimination of ERK-dependent pain hypersensitivity (17). In cultured neurons, patient antibodies cause a reduction in DPPX and Kv4.2 membrane proteins, while increasing the activity of enteric neurons. However, these effects are reversible upon antibody removal (7, 18). Anti-DPPX antibodies target the DPPX protein, which is highly expressed in the enteric nervous plexus (Auerbach/Meissner plexus), causing dysfunction of enteric potassium channels (Kv4.2). This leads to prodromal gastrointestinal symptoms and may be triggered by a preceding infection, initiating a systemic autoimmune response. Ultimately, the antibodies can cross the blood-brain barrier and spread to the central nervous system, inducing increased neuronal excitability and associated neuropsychiatric symptoms (19, 20). DPPX is highly expressed in the enteric myenteric plexus, and the antibodies may affect baroreflex function via the vagus-solitary tract nucleus pathway, leading to poor orthostatic tolerance. Tobin et al. described among the 20 patients with DPPX-IgG positivity, multifocal neurological symptoms (such as amnesia and brainstem dysfunction), central hyperexcitability (such as myoclonus), and autonomic dysfunction (such as gastrointestinal symptoms) were the main clinical manifestations. Among the 11 patients who received immunotherapy, 7 showed symptom improvement (7). 63.5% of patients with DPPX-AE exhibit central nervous system hyperexcitability as a prominent feature, manifesting as myoclonus, tremors, seizures, rigidity/stiffness, and exaggerated startle responses to auditory or tactile stimuli (which may be accompanied by myoclonus and spasms of the head, neck, or trunk) (8, 20, 21). Tremor is an involuntary, rhythmic movement involving joint axes (22). Tremor may reflect persistent inflammation of the cerebellar-thalamo-cortical circuit or postsynaptic receptor downregulation (23). Tremor has not yet been defined as a core characteristic manifestation of antibody-mediated diseases; however, previous studies suggest it may be associated with certain cases of NMDAR, LGI1 or DPPX-AE (6, 7, 24, 25). Homonymous hemianopia stemming from identical visual field defects in both eyes is typically caused by occipital lobe infarction (26). Corticosteroids can upregulate the expression of transforming growth factors, which increases IL-10 levels, and reduce the expression of immunoglobulins and Fc receptors secreted by macrophages, thereby inhibiting inflammation and damage to the blood-brain barrier (12). Autoantibodies against neurons in the bloodstream may enter the brain through temporary breaches in the blood-brain barrier or via Fc receptor-mediated endocytosis, thereby increasing blood-brain barrier permeability, promoting the production of inflammatory factors, and stimulating the migration of immune cells within the central nervous system (27). However, it is currently uncertain whether this mechanism can facilitate antibody entry into the central nervous system during AE. The disease tends to progress insidiously, generally peaking at around 8 months, at which point all patients can be detected with IgG DPPX antibodies (7). Seronegative cases hold significant clinical importance, as they differ from antibody-positive autoimmune encephalitis, indicating that their insidious nature and heterogeneity require further differentiation. Patients with early-stage DPPX-AE respond well to immunotherapy, including sequential steroid treatment (IVMP) and oral steroids. Recurrence often occurs as steroids are gradually tapered, necessitating long-term immunotherapy to maintain prognosis (6–8, 18). The focus on AE should not be limited to the current understanding of antibody subtypes; rather, it should be viewed as a more specific disease syndrome. As more antibodies are discovered, this challenge is likely to persist. Overall, through this case report, we offer some useful recommendations. Future advancements in medicine may lead to the development of a more unified disease-specific biomarker to assist in more accurate diagnosis, prevention, and disease monitoring.

Efgartigimod is a novel Fc receptor-targeted biologic that works by blocking the interaction between FcRn and IgG, thereby accelerating the catabolism of circulating IgG (28), its mechanism of action is particularly suited to IgG-mediated autoimmune diseases (29). Antibody-negative probable AE(ANPRA) may involve undetectable pathogenic IgG antibodies or non-classical autoantibodies. Thus, Efgartigimod accelerates clearance of all IgG subclasses (including potential pathogenic autoantibodies below detection thresholds) by competitively blocking FcRn-mediated recycling (30, 31). This offers a broad immunomodulatory effect against antibody-mediated pathology even when specific antibodies aren’t identified. In recent years, this drug has demonstrated remarkable efficacy in the treatment of myasthenia gravis (MG) and chronic inflammatory demyelinating polyneuropathy (32). In the clinical study NCT04818671, which primarily focused on MG, 12 patients with coexisting central nervous system autoimmune diseases were included, three of whom had anti-NMDAR encephalitis. After treatment with efgartigimod, two patients achieved an improvement of ≥2 points in mRS scores, and CSF antibody titers decreased by more than 50%, suggesting a potential central penetration effect on antibodies (33). Ofatumumab, a fully human anti-CD20 monoclonal antibody, binds to the CD20 molecule on the surface of B cells and induces B cell death through antibody-dependent and complement-dependent cytotoxicity pathways (34). ANPRA could involve B-cell-driven inflammation (T-cell cytotoxicity, cytokine release) without detectable antibodies. Ofatumumab depletes CD20+ B-cells (naïve/memory), disrupting antigen presentation, T-cell activation, and potential antibody production (35). Here have been successful cases in which ofatumumab was applied to DPPX-AE (36), indicating that it may be a potential treatment option for this condition. However, ANPRA heterogeneity implies mechanisms may extend beyond antibody-mediated pathology (such as cytotoxic T-cells), which FcRn/CD20 agents don’t fully address (37, 38). The use of these two drugs in DPPX-AE remains exploratory, and this case provides important practical data for advancing the field.

## 4 Patient perspective

In communication with the patient, it was explained that currently, DPPX-AE is a relatively rare disease. Abnormal immune system attacks on the brain can lead to symptoms such as headaches and tremors, and may later be accompanied by gynecological issues or gastrointestinal discomfort. A very small number of patients should be cautious of tumor risks. Especially for diseases related to the autoimmune system, the diagnosis and treatment process is prolonged, and symptoms may fluctuate during this period. However, the conversion of antibodies to negative indicates that immunotherapy is effective. We are confident and determined to win this long-term battle and will approach any new developments with a rational perspective to analyze and resolve the issues. The patient and their family have gone from a lack of understanding of the disease to providing positive feedback during monthly follow-ups. Following the second recurrence, the patient and his family experienced significant distress, and the treatment plan lacked proper coordination, resulting in an unsatisfactory outcome during the second hospitalization. Despite challenges, we strive for the best possible outcome.

## 5 Materials and methods

### 5.1 Antibody testing using cell-based assay

The anti-DPPX antibodies were identified through a cell-based assay (CBA). The CBA employed the Human Epithelioma-2 (HEp-2) cell line. The HEp-2 cells were cultured following standard procedures to ensure appropriate expression of the target antigens relevant for the detection of anti-DPPX antibodies. The CBA utilized in this study was a commercial assay. However, due to confidentiality agreements and restrictions from the commercial provider, the specific name of the test kit cannot be disclosed. Regarding the antibody isotype detected, the focus was on the IgG isotype of anti-DPPX antibodies as it holds significance in understanding the immune response related to the condition under investigation. It should be noted that in the testing process, a positive control was not included. This is due to the fact that we did not obtain a suitable positive control sample with the exact characteristics required for that particular assay device. Instead, a negative control was employed, which consisted of a normal human serum sample that had been verified through multiple pre-tests to be free of anti-DPPX antibodies. This negative control played a crucial role in ensuring the baseline performance and accuracy of the assay. Overall, despite the limitations in terms of the positive control, alternative validation methods and quality assurance steps were taken to ensure the reliability of the assay results and the conclusions drawn from this antibody testing approach.

## Data Availability

The original contributions presented in the study are included in the article/supplementary material. Further inquiries can be directed to the corresponding author.
